# Predicting direct protein interactions from affinity purification mass spectrometry data

**DOI:** 10.1186/1748-7188-5-34

**Published:** 2010-10-29

**Authors:** Ethan DH Kim, Ashish Sabharwal, Adrian R Vetta, Mathieu Blanchette

**Affiliations:** 1 McGill Centre for Bioinformatics, McGill University, Quebec, Canada; 2 Department of Computer Science, Cornell University, NY, USA; 3 Department of Mathematics and Statistics, McGill University, Quebec, Canada

## Abstract

**Background:**

Affinity purification followed by mass spectrometry identification (AP-MS) is an increasingly popular approach to observe protein-protein interactions (PPI) *in vivo*. One drawback of AP-MS, however, is that it is prone to detecting indirect interactions mixed with direct physical interactions. Therefore, the ability to distinguish direct interactions from indirect ones is of much interest.

**Results:**

We first propose a simple probabilistic model for the interactions captured by AP-MS experiments, under which the problem of separating direct interactions from indirect ones is formulated. Then, given idealized quantitative AP-MS data, we study the problem of identifying the most likely set of direct interactions that produced the observed data. We address this challenging graph theoretical problem by first characterizing signatures that can identify weakly connected nodes as well as dense regions of the network. The rest of the direct PPI network is then inferred using a genetic algorithm.

Our algorithm shows good performance on both simulated and biological networks with very high sensitivity and specificity. Then the algorithm is used to predict direct interactions from a set of AP-MS PPI data from yeast, and its performance is measured against a high-quality interaction dataset.

**Conclusions:**

As the sensitivity of AP-MS pipeline improves, the fraction of indirect interactions detected will also increase, thereby making the ability to distinguish them even more desirable. Despite the simplicity of our model for indirect interactions, our method provides a good performance on the test networks.

## Background

Understanding the organization of protein-protein interactions (PPIs) as a complex network is one of the main pursuits in proteomics today. With the help of high-throughput experimental techniques, a large amount of PPI data has recently become available, providing us with a rough picture of how proteins interact in biological systems. However, the interaction data from these high-throughput experiments suffer from low resolution as compared to data from low-throughput technologies such as protein co-crystallization, and to make matters worse, they are prone to problems including relatively high error rates and protocol-specific biases. Therefore, inferring the direct, physical PPI network from high-throughput data remains a challenge in systems biology.

The leading technologies for identifying PPIs are Yeast 2-Hybrid (Y2H) [1,2] and Affinity Purification followed by Mass Spectrometry (AP-MS) [3-6]. Due to the ability to perform *in vivo *at biologically reasonable expression levels, as well as the ability to detect protein complexes with fewer false-positives [6], AP-MS approaches have become increasingly popular, although their throughput is lower than Y2 H approaches. In an AP-MS experiment, a protein of interest (the *bait*) is tagged and expressed *in vivo*. The bait is then immuno-precipitated (IP), together with all of its interacting partners (the *preys*), and finally, preys are identified using mass spectrometry. For a more detailed overview of the technique, see [6,7]. Like Y2 H and other high-throughput experimental methods, however, AP-MS suffers from experimental noise. A number of approaches have been proposed to separate true interactions from false-positives. These approaches mostly focus on reducing false-positives due to protein misidentification from MS data [8-10], on detecting contaminants [11], or a combination of both [7,12-16]. These methods often make use of the *guilty-by-association *principle, and quantify the confidence level of an interaction by considering alternative paths between two protein molecules. In this context, authors say that a true interaction between bait *b *and prey *p *is a true positive if, at some point in the set of cells considered, there exists a complex that contains both *b *and *p*. We note that as the sensitivity of the AP-MS methods improves and the stability of the complexes that can be detected decreases, the transience of detectable interactions will increase, to a point where, eventually, every protein may be shown to marginally interact with every other protein.

A key property of AP-MS approaches is that a significant number of the co-purified prey proteins are in fact *indirect *interaction partners of the bait protein, in the sense that they do not interact physically and directly with the bait, but interact with it through a chain of physical interactions involving other proteins in the complex. Therefore, it is critical, when interpreting AP-MS-derived PPI networks, to understand the meaning of the term "interaction". Although not designed to identify physical interactions, AP-MS experiments produce data that may allow separating direct physical interactions from indirect ones. This is the problem we consider in this paper: *given the results of a set of AP-MS experiments, filtered for protein misidentifications and contaminants, how can we distinguish direct (physical) interactions from indirect interactions? *Note that since the false-positive filtering methods listed above consider indirect interactions as true-positives, they cannot be used to address this problem. Gordân *et al*. [17] study the related problem of distinguishing direct vs. indirect interactions between transcription factors (TF) and DNA. While the objective of their study is similar to ours, their method makes use of information specific to TF-DNA interactions (e.g. TF binding data, motifs from protein binding microarrays), and thus is not immediately applicable to the problem on general PPI networks. In fact, to our knowledge, no existing approach seems directly applicable.

This paper is organized as follows. We first describe the mathematical modelling of an AP-MS experiment and introduce an algorithmic formulation of the problem. We then describe an overview of our method, which is based on a collection of graph theoretic approaches that succeeds at inferring a large fraction of the network nearly exactly, followed by a genetic algorithm that infers the remainder of the network. The accuracy of the proposed method is assessed using both biological and simulated PPI networks. Finally, we apply our algorithm to the prediction of direct interactions based on a large set of AP-MS PPI data in yeast [18]. Our work opens the way to a number of interesting and challenging problems, and the results obtained indicate that useful inferences can be made despite the simplicity of our modelling.

## Results

Because the main contribution of this paper is methodological, we start by giving an overview of the approach developed before detailing the results obtained.

Throughout this work, we make the assumption that appropriate methods have been used to reduce as much as possible protein misidentifications and contaminants, in such a way that all interactions detected are either direct or indirect interactions. Our task is to separate the former from the latter. To avoid confusion, we note that false-positives (resp. false-negatives) henceforth refer to falsely detected (resp. undetected) *direct *interactions inferred by our algorithm.

### Mathematical modelling of AP-MS data

We first describe a simple model of the AP-MS PPI data that shall be used throughout this paper. Although admittedly rather simplistic, our model has the benefit of allowing the formulation of a well-defined computational problem.

**Figure 1:**
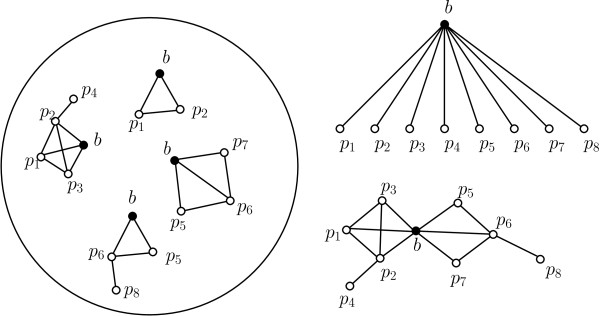
**Indirect interactions in AP-MS PPI data**. In a cell, multiple copies of a bait protein *b *are expressed, and interact (directly or indirectly) with other proteins *p*_1_, ..., *p*_8 _(left); After the pull-down on the bait *b*, MS detects all prey proteins, including indirect interaction partners (top right); The direct interaction network should, however, contain only the edges between direct interaction partners (bottom right).

Let *G_direct _*= (*V, E_direct_*) be an undirected graph whose nodes *V *represent the set of proteins, and edges *E_direct _*represent direct (physical) interactions between the proteins. Let *N*(*b*) = {*p *∈ *V *: (*b, p*) ∈ *E_direct_*} be the set of direct interaction partners of protein *b*. We model the physical process through which PPIs are identified in an AP-MS experiment as follows. If a bait protein *b *is in contact with a direct interaction partner *p *∈ *N*(*b*), the IP on *b *will pull *p *down, which will then be identified through mass spectrometry. In addition, if *p *interacts with *p' *∈ *N*(*p*) at the same time as it interacts with *b*, protein *p' *may also be pulled down by the IP on *b*, although the two proteins only *indirectly *interact. In general, any protein *x *that is connected to *b *by a series of simultaneous direct interactions may be pulled down by *b*. As a result, all interaction partners of *b *(direct or indirect) will be identified together. Figure 1 depicts an example of this effect. In order to distinguish direct physical interactions from indirect ones, the availability of *quantitative *AP-MS data is helpful.

Although quantitative AP-MS remains at its infancy, prey abundance can be estimated fairly accurately using approaches such as the peptide count [19], spectral count [20], sequence coverage [21], and protein abundance index [22]. Combined with increasing accuracy and sensitivity of mass spectrometers, these methods are becoming more reliable. Throughout the discussions in this paper, we assume that this quantitative data is available to us.

The strength of a physical interaction can be measured by the energy required to break it. Let *A*(*b, x*) denote the abundance of a prey protein *x *obtained by IP on bait *b*, and let *χ*(*p*_1_, *p*_2_) denote the number of pairs of molecules *p*_1 _and *p*_2 _that interact directly in the cells considered. When there are more than two interaction partners, we let *χ*(*p*_1_, *p*_2_, ..., *p_k_*) denote the number of copies of complexes simultaneously containing *p*_1_, *p*_2_, ..., *p_k_*. Since protein interactions may be disrupted by the purification process, we expect *A*(*b, x*) to be correlated with the strength of the interaction between *b *and *x*. Thus, we assume that a direct interaction between a pair of individual proteins *b *and *x *survives the purification process with probability $\hat{p}(b,\;x)$, and breaks with probability $1-\hat{p}(b,\;x)$. Then the amount of protein *x *obtained from the pull-down on *b *would be

$$A(b,x)\propto \chi (b,x)\cdot \hat{p}(b,x).$$

Consider now a system of three proteins *b, x, x'*, where (*b, x*) and (*x, x*^'^) form direct interactions but *b *and *x' *do not interact directly. Then, $A(b,x^{'})\propto \chi (b,x,x^{'})\cdot \hat{p}(b,x)\cdot \hat{p}(x,x^{'})$. In general, the amount of protein *x *that will be obtained upon pull-down of *b *will be proportional to the probability that *b *and *x *remain connected after each edge (*u, v*) ∈ *E_direct _*is broken with probability $\hat{p}$(*u, v*). Our goal is then to infer *G_direct _*from the set of observed abundances *A*(*x, y*). In this paper, we make the following simplifying assumptions:

1. All direct interactions (*u, v*) ∈ *E_direct _*survive with the uniform probability $\hat{p}$, and fail independently with probability 1 - $\hat{p}$.

2. All possible direct interactions take place at the same time, irrespective of the presence of other interactions, and with the same frequency.

Although these assumptions are clearly unrealistic, they provide a useful starting point for separating direct interactions from indirect ones (see Discussion for possible relaxation of these assumptions). Despite its simplicity, our mathematical modelling of AP-MS does fit existing biological data reasonably well (see Model validation). We note that Asthana *et al*. [23] have proposed a probabilistic graph model that is similar to ours. However, their model measures the likelihood of a protein's membership in a protein complex, and thus is not applicable to our problem.

### Problem formulation

We are now ready to formulate the algorithmic problem addressed in this paper. We henceforth consider the (unknown) direct interaction network *G_direct _*as a probabilistic graph, where each edge in *G_direct _*survives the AP-MS process with probability $\hat{p}$, and fails otherwise. Let ${\tilde{G}}_{direct}$ be a random graph obtained from *G_direct _*by removing edges in *E_direct _*independently with probability 1 - $\hat{p}$. Then, define $P_{G_{direct}}(u,\;v)$ to be the probability that vertices *u *and *v *remain connected (directly or indirectly) in ${\tilde{G}}_{direct}$:

$$P_{G_{direct}}(u,\;v)=\text{Pr}\left[\text{there exists at least one path from }u\;\text{to }v\;\text{in }{\tilde{G}}_{direct}\right].$$

**Figure 2:**
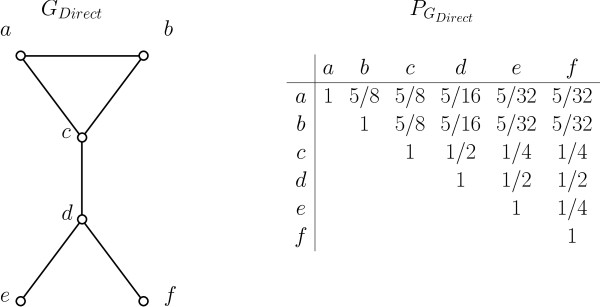
**Example of a direct interaction network and its connectivity matrix**. An example of a direct interaction network *G_Direct _*(left) with its connectivity matrix $P_{G}{}_{{}_{Direct}}$ (right) calculated with $\hat{p}=\frac{1}{2}$. Assuming each edge of *G_Direct _*survives with probability $\hat{p}$, the probability of connectivity between each pair of protein can be estimated via sampling of the probabilistic network.

We call $P_{G_{direct}}$ the *connectivity matrix *of *G_direct_*. See Figure 2 for an example of a direct interaction network and its connectivity matrix. Although $P_{G_{direct}}(u,\;v)$ can be estimated from *G_direct _*by straight-forward Monte Carlo sampling, its exact computation (known as two-terminal network reliability problem [24,25]) is #P-Complete [24], and so is its approximation within a relative error of *ϵ *[26].

A set of AP-MS experiments where all proteins have been tagged and used as baits yields an approximation of *A*(*x, y*) for all pairs of proteins (*x, y*), which can be transformed into an estimate *M*(*x, y*) of $P_{G_{direct}}(x,\;y)$ through appropriate normalization. We are thus interested in inferring *G_direct _*from *M*:

EXACT DIRECT INTERACTION GRAPH FROM CONNECTIVITY MATRIX (E-DIGCOM)

**Given: **A connectivity matrix *M_n × n_*

**Find: **A graph *G *= (*V, E*) such that *P_G_*(*u, v*) = *M*(*u, v*) for each *u, v *∈ *V*.

In a more realistic setting, the connectivity matrix *M *would not be observed precisely, and the E-DIGCOM problem may not admit a solution. We are thus interested in an approximate, optimization version of the problem:

APPROXIMATE DIRECT INTERACTION GRAPH FROM CONNECTIVITY MATRIX (A-DIGCOM)

**Given: **A connectivity matrix *M_n× n _*and a tolerance level 0 ≤ *δ *≤ 1

**Find: **A graph *G *= (*V, E*) such that the number of pairs (*u, v*) ∈ *V × V *such that |*P_G_*(*u, v*) **- ***M*(*u, v*)| **≤ **δ is maximized.

Note that although the computational complexity of the DIGCOM problems is currently unknown, the fact that simply verifying a candidate solution is #P-Complete suggests that the problem may be hard and may not belong to NP - candidate solutions to problems in NP can, by definition, be verified in polynomial time, whereas #P is widely believed to require super-polynomial time computations. Related problems include network design problems that have been studied extensively in the computer networking community. For example, one related, but different, problem is to choose a minimal set of edges over a set of nodes so that the resulting network has at least the prescribed all-pairs terminal reliability; various algorithms including branch-and-bound heuristics [27] and genetic algorithms [28,29] have been proposed.

### Algorithm overview

Our algorithm for the A-DIGCOM problem has three main phases outlined here and detailed in Methods.

Phase I. We start by identifying, based on the connectivity matrix *M*, vertices from *G_direct _*with low degree, together with edges incident to them. As most PPI networks exhibit the properties of scale-free networks [30], this resolves the edges incident to a significant portion of the vertices (~75% in our networks; see below).

Phase II. At the other end of the spectrum, *G_direct _*contains densely clustered regions (cliques or quasi-cliques), possibly corresponding to protein complexes. We use a heuristic to detect these dense regions from the connectivity matrix *M*.

Phase III. To infer the remainder of the network, we use a genetic algorithm. This highly customized genetic algorithm makes use of the findings from the previous two steps in order to dramatically reduce the dimension of the problem space, and to guide the mating process between parent candidates to create good offspring solutions.

In what follows, we highlight the main theoretical results on which these three phases rely. Details are given in Methods and proofs in Appendix.

#### I-a. Finding cut edges

A *cut edge *in a graph *G *is an edge (*u, v*) whose removal would result in *u *and *v *belonging to two distinct connected components (e.g. edge (3,4) in Figure 2). The following theorem allows the identification of all cut edges based on the connectivity matrix *P_G_*.

**Theorem 1**. *A pair of vertices u and v from V forms a cut edge in G if and only if the following two conditions hold*.

*(i) *$P_{G}(u,\;v)=\hat{p}$

*(ii) V can be partitioned into V *= *V_u _*∪ *V_v_ , where V_u _*= {*x *∈ *V *: *P_G_*(*x, u*) *≥ P_G_*(*x, v*)} *and V_v _*= {*x *∈ *V *: *P_G_*(*x, u*) <*P_G_*(*x, v*)} , *such that *∀*s *∈ *V_u _and *∀*t *∈ *V_v_*, $P_{G}(s,\;t)=P_{G}(s,\;u)\cdot \hat{p}\cdot P_{G}(v,\;t)$.

The above theorem immediately provides an efficient algorithm, requiring time *O*(|*V*|^2^), to test whether a pair of vertices forms a cut edge. Observe that removing a cut edge (*u, v*) from a connected graph allows us to decompose the graph into two connected components (subgraphs induced by *V_u _*and *V_v_*, respectively), and the probability of connectivity between every pair of vertices in *V_u _*(*V_v_*, resp.) remains the same after removing (*u, v*). Therefore, the submatrices that correspond to *V_u _*and *V_v _*can be treated as independent subproblems, and one can recursively detect cut edges in the remaining subproblems. Note that if the input graph is assumed to be a tree, such a recursive algorithm would identify the entire graph exactly. On the other hand, PPI networks are sparse in general, and contain many cut edges and degree-1 vertices. As a result, this algorithm allows a significant simplification of our problem by identifying all cut edges.

#### I-b. Finding degree-2 vertices

We now consider the problem of identifying degree-2 vertices from the connectivity matrix *M*. After degree-1 vertices, which are identified in the previous step, they constitute the next most frequent vertices in the biological networks we studied. While we do not have a full characterization of these vertices, the following theorem gives a set of necessary conditions.

**Theorem 2**. *Let s be a degree-2 vertex in G such that N*(*s*) = {*u, v*}. *Then, the following three conditions must hold*.

(*i*) **Low connectivity: ***for each t ∈ V*, $P_{G}(s,t)<2\hat{p}-{\hat{p}}^{2}$.

(*ii*) **Neighborhood: ***for each t ∈ V *- {*s*, *u*, *v*}, *P_G _*(*s*, *t*) <*P_G _*(*s*, *u*) = *P_G _*(*s*, *v*).

(*iii*) **"Triangle" inequality: ***for each t ∈ V *- {*s*, *u*, *v*}, *P_G _*(*s*, *t*) <*max*{*P_G _*(*u*, *t*), *P_G _*(*v*, *t*)}.

These necessary conditions allow us to rule out vertices that cannot be of degree 2, and give rise to a *O*(|*V*|^2^) heuristic for predicting degree-2 vertices (see Algorithm 1 in Methods). In practice, our studies have shown that vertices satisfying these conditions while having degree higher than two are extremely rare (see below).

#### II. Detecting densely connected regions

We now turn to the problem of finding densely connected regions in the network. These regions may correspond to protein complexes, where tagging any one of the members of the complex results in the identification of all other members of the complex with high probability. While correctly predicting the physical interactions within each complex is a difficult task, separating these dense regions from the remainder of the network is essential to improving the accuracy of the genetic algorithm (part III).

Based on the connectivity matrix *M*, our algorithm identifies (possibly overlapping) clusters of proteins of size at least *k *such that, for every pair *u, v *in each cluster, *M*(*u, v*) ≥ *t_k _*for some threshold *t_k_*. For appropriately chosen values of *k *and *t_k _*(see Methods), the set of clusters found corresponds to cliques in *G_direct _*with high accuracy (see below).

The dense regions discovered at this phase provide us (1) the set of edges within each dense region; and (2) sparse cuts between disjoint dense regions. The edge set within each cluster will be used in the initial candidates for the genetic algorithm, whereas the cuts defined by the clusters will be used as crossover points during the crossover operation in the genetic algorithm.

#### III. Cut-based genetic algorithm

To predict the remaining section of the network, we use a customized genetic algorithm that aims at finding an optimal solution to the A-DIGCOM problem. We first devise a solution to a generalization of the A-DIGCOM problem, and then show how the results of parts I and II of the algorithm are used to improve performance.

Genetic algorithms have been shown to be an effective family of heuristics for a wide variety of optimization problems [31], including network design under connectivity constraints [28,29]. A genetic algorithm models a set of candidate solutions as individuals of a population. From this population, pairs of promising candidate solutions are mated, and their off spring solutions inherit properties of the parents with some random mutations. Over generations, this process of natural selection improves the fitness of the population.

The A-DIGCOM problem is a hard optimization problem, because (i) the size of the search space is huge - $2^{\left(\begin{array}{l} n\\ 2 \end{array}\right)}$ for a graph of size *n*, and (ii) there is no known polynomial-time algorithm to evaluate a proposed candidate solution (i.e. compute *P_G _*from *G*). For these reasons, a straight-forward genetic algorithm implementation failed to produce satisfactory results (data not shown). Instead, we use a more sophisticated approach by making use of the results obtained in previous sections in order to reduce the search space and to guide the mating operations for more effective search. Details are given in Methods.

### Model validation

In order to test our approach, we first sought to validate our model of AP-MS indirect interactions. To this end, we used one of the most comprehensive AP-MS-based networks published to date on yeast, obtained by Krogan *et al*. [18]. The dataset reports the Mascot score [32] and the number of peptides detected for each bait-prey pair (peptide count). The complete set of interactions reported contains 2186 proteins and 5496 interactions (Krogan *et al*. Table S six); we call the resulting network *G_KroganFull_*. The authors identified a subset of these interactions as high-confidence, based on their Mascot scores (Krogan *et al*. Table S five). We call this set of high-confidence interactions *G_KroganHigh_*; this network consists of 1210 proteins and 2357 interactions. We expect that *G_KroganHigh _*is relatively rich in direct interactions, whereas the complete set of interactions *G_KroganFull _*consists in part of indirect interactions.

Considering *G_KroganHigh _*as a direct interaction network, we simulated Monte Carlo sampling to estimate $P_{G_{Krogan\;High}}$, using $\hat{p}=0.5$ and 50,000 samples, which yields a 95% confidence interval of size at most 0.007 on each $P_{G_{Krogan\;High}}(u,\;v)$ entry. Next, we normalized the peptide counts of the interactions in *G_KroganFull _*using protein lengths (See Methods). We then compared $P_{G_{Krogan\;High}}$ to the normalized peptide counts of the interactions in *G_KroganFull_*. We expect that a significant fraction of low-confidence interactions in *G_KroganFull _*- *G_KroganHigh _*are likely to be indirect interactions. If our model is correct, their peptide counts should then be correlated with the corresponding entries in $P_{G_{Krogan\;High}}$. Indeed, the positive linear correlation between the predicted connectivity $P_{G_{Krogan\;High}}$ and the observed normalized peptide counts is very significant (regression p-value of 8.17 × 10^-11^, Student *t*-test; see Additional file 1). Furthermore, this correlation is strongest when $\hat{p}\approx 0.5$, as compared to $\hat{p}=0.3$ or 0.7, justifying the use of this value in our subsequent analyses.

### Accuracy of the prediction algorithm

The ideal validation of the accuracy of our algorithm would involve (i) constructing a connectivity matrix *M *using actual quantitative AP-MS data; (ii) predict direct interactions based on *M *using our algorithm; and then (iii) comparing our predictions to experimentally generated direct interaction data. Yeast 2-Hybrid (Y2H) experiments are less prone to detect indirect interactions than are AP-MS methods, and several large-scale efforts have been reported [2,33,34]. Unfortunately, for a number of technical reasons, the overlap between AP-MS PPI networks and Y2H networks remains very small [35]. As a consequence, Y2 H data cannot be used directly to validate predictions made on AP-MS data. Instead, we had to rely on partially-synthetic data set, where an actual network of high-quality Y2 H interactions is assumed to form the direct interaction graph, and a connectivity matrix is generated from it using Monte Carlo sampling, under our model. Two sets of Y2 H interactions were used: (i) *G_Y u _*is the network constructed from the gold standard dataset of Yu *et al*. [35]. This network consists of 1090 proteins and 1318 interactions with high confidence of direct interactions; (ii) *G_DIP _*is the core, high-quality, physical interaction network of yeast, available at DIP database, version 20090126CR [36], consisting of 1406 proteins and 1967 interactions. These biological networks were complemented with two artificial 1000-vertices networks. The first was generated using the preferential attachment model (PAM) [30]. For the second, we used the duplication model (DM) [37], which, in contrast to the PAM, generates graphs containing several dense clusters. The resulting artificial "direct" interactions graphs are called *G_PAM _*and *G_DM _*and contain 1500 ~ 2000 interactions each. We then used the Monte Carlo sampling approach described above to estimate the connectivity matrices $P_{G_{\;Yu}},P_{G_{DIP}},P_{G_{PAM}}$, and $P_{G_{DM}}$. These will form the input to our inference algorithm, whose output will then be compared to the corresponding direct interaction graph. It is important to note that these input matrices are not perfectly accurate and may contain sampling errors. However, it is easy to bound the size of the errors with high probability and use it as a tolerance level within our algorithm. We also note that the results presented in this section only aim at evaluating the performance of the inference algorithm on input data that was generated exactly according to our probabilistic model. As such, the error rates reported may be considered as lower bounds for those on actual biological data. An assumption-free evaluation in provided later in this section.

#### Identification of weakly connected vertices

Theorem 1 provides an efficient algorithm that guarantees the identification of all cut edges, provided that the given connectivity matrix is precise. We say that a vertex *v *is a *1-cut vertex *if *all *edges incident on *v *are cut edges. By applying Theorem 1 recursively to detect cut edges and decomposing the graph into two connected components, we can detect and remove all 1-cut vertices from the input connectivity matrix.

Table 1 (i) reports the number of 1-cut vertices that are detected by the recursive algorithm from Theorem 1. In both the Yu and the DIP network, 1-cut vertices constitute approximately 50% of the network, and identifying them allows a significant reduction in the problem size. We note that the inaccuracies in the input connectivity matrices could, in principle, have introduced errors in the detection of cut edges. However, this rare event was never observed on any of our networks.

**Table 1:** Performance of detecting weakly connected vertices

		(i) 1-cut vertices				(ii) Degree 2 vertices				
				
Network	Total	real	**pred**.	FDR(%)	FNR(%)	real	**pred**.	FDR(%)	FNR(%)	Remaining
Yu	1090	552	552	0	0	195	207	7.7	2.05	331
DIP	1406	656	656	0	0	309	326	5.82	0.64	424
PAM	1000	457	457	0	0	351	363	3.58	0.28	180
DM	1000	323	323	0	0	117	126	11.9	5.12	551

Number of vertices detected as (i) 1-cut vertices, and (ii) degree 2 vertices in the real network and the predicted network. False discovery (FDR) and false negative (FNR) ratios are given in percentage. Remaining: the number of vertices remaining after identifying 1-cut vertices and degree 2 vertices.

Algorithm 1 (see Methods) guarantees to efficiently identify all degree-2 vertices (again, provided that the connectivity matrix is known), but may also incorrectly flag some higher-degree vertices. As seen in Table 1 (ii), nearly all degree-2 vertices were identified, with a low false-discovery rate ranging from 6 to 9%. Moreover, the false-positives incorrectly detected as degree-2 vertices indeed had small degrees, and their predicted neighbors were mostly correct (but incomplete) predictions. Flagging degree-2 vertices reduces the problem size further by 15 to 36%.

After repeatedly detecting and removing 1-cut vertices and degree-2 vertices from the problem space, the edges adjacent to approximately 70% of the vertices are detected with very low error rate. The remaining vertices only constitute approximately 30% of the original network. We call this remaining subset the *hard core *of the connectivity matrix. Because it is more densely connected than the rest of the network, the topology of hard core is more difficult to reconstruct.

Running our algorithm on the PAM simulated data yields similar resolution and error rate as on the Y2 H networks. However, our DM network is found to be less amenable to these strategies, leaving 55% of vertices unresolved and resulting in an error rate approximately twice that seen for other networks. This is simply due to the fact that networks generated by the duplication model do not contain as many 1-cut vertices or degree 2 vertices when compared to other networks, including the biological Y2 H networks.

#### Identification of dense regions

Our dense region detection algorithm aims at identifying all edges that belong to a *k*-clique in *G_Direct_*, for a given value of *k*. We report the accuracy of the algorithm in Table 2. As expected, our algorithm achieves extremely high sensitivity for clique edges. However, the false-discovery rate is quite high, especially for smaller values of *k *(e.g. *k *= 5). This is due to the fact that distinguishing a 5-clique from, say, a quasi-clique of size 7 is extremely difficult, causing false-positive predictions. We note however that these erroneous predictions are mostly inconsequential, as the intra-cluster topology of each dense region shall only be used in generating the initial candidate solutions for the genetic algorithm.

**Table 2:** Performance of quasi-clique predictions

Network	*k *= 7				*k *= 6				*k *= 5			
			
	real	**pred**.	FD(%)	FN(%)	real	**pred**.	FD(%)	FN(%)	real	**pred**.	FD(%)	FN(%)
Yu	42	54	22.22	0	66	104	36.53	0	146	308	55.19	5.48
DIP	0	42	100	0	86	112	26.79	4.65	184	266	35.34	6.52
PAM	0	31	100	0	0	45	100	0	0	96	100	0
DM	254	346	28.32	2.36	194	267	29.21	2.57	488	718	34.96	4.30

Number of edges that belong to maximal cliques of size *k*. Real: actual number of edges that belong to maximal cliques of size *k*; pred: predicted number of maximal *k*-clique edges; FD(false discovery ratio): percentage of false-positives in the predicted set; FN(false negative ratio): percentage of false-negatives in the real set.

#### Cut-based genetic algorithm

The various parameters of the genetic algorithm (population size, mate selection probability, mutation rate, etc.) were optimized for the running time and accuracy of the solution based on *G_Yu_*. Although our genetic algorithm could in principle be used on any connectivity matrix, running it on the full matrix of > 1000 proteins is impossible: the search space is huge, and the amount of time required to evaluate the fitness of a given candidate solution is too large. However, as discussed previously, applying first the 1-cut and degree-2 vertex detection algorithms significantly reduces the problem size and makes it accessible to our genetic algorithm. Table 3 (i) reports the accuracy of the genetic algorithm predictions on the hard core of each connectivity matrix. We note that since the network to be inferred is relatively highly connected, the problem is significantly more difficult than the identification of 1-cuts and degree-2 vertices. Indeed, the false-discovery and false-negative rates range from 35% to 55% for most datasets. For a comparison, an algorithm that would pick edges randomly would achieve 98.75% false-discovery and false-negative rates. Combining the three phases of the algorithm, the overall error rate obtained on each data set ranges from 10 to 20% false-discovery and false-negative rates, except for the DM data set, which fares considerably worse, for the reasons explained earlier.

**Table 3:** Performance of genetic algorithm and overall algorithm

Network	(i) reduced network				(ii) overall network			
		
	real	**pred**.	FDR(%)	FNR(%)	real	**pred**.	FDR(%)	FNR(%)
Yu	563	552	43.65	44.76	1318	1390	14.96	10.31
DIP	931	890	35.50	38.34	1967	2041	17.34	14.23
PAM	473	421	49.88	55.39	1538	1462	16.14	20.28
DM	1138	1295	43.39	35.58	1869	1804	32.81	35.15

(i) Performance of the genetic algorithm on the reduced network obtained from removing 1-cut vertices and degree-2 vertices. (ii) Overall performance of the combined prediction pipeline on the complete connectivity matrix. Real and predicted describe the number of edges in the real and predicted networks, respectively.

To the best of our knowledge, there has been no other efforts to solve the DIGCOM problems (neither exact, nor approximate version). We thus compared our approach to a simple hill climbing search algorithm on the Yu *et al*. data set (see Methods). We let this algorithm run over several days (as opposed to few hours spent using our approach), with multiple restarts, and discovered that it provides very poor sensitivity and specificity (see Table 4 for the best results obtained). This is not surprising since the hill climbing method is highly dependent on the initial solution (in this case, a spanning tree chosen randomly based on the connectivity matrix) and the search space is simply too large to exhaustively search for the a good initial solution. We also tested the hill-climbing approach in the same setting as the genetic algorithm, i.e. combining it with the 1-cut edges and degree-2 vertices detection algorithms. Here, the modified hill-climbing approach showed a better sensitivity and specificity than the pure hill-climbing approach, but still performed much worse than our genetic algorithm (Table 4). Furthermore, the improvement over the pure hill-climbing approach was mostly due to the high sensitivity and specificity of our algorithm for detecting weakly connected vertices.

**Table 4:** Comparison of our method to simple hill-climbing approach

Network	Real	Predicted	FDR(%)	FNR(%)
(i) Hill-climbing	1318	2108	86.67	78.68
(ii) Hill-climbing + weakly conn. nodes	1318	1517	43.57	35.05
(iii) Our approach (GA)	1318	1390	14.96	10.31

Comparison of our method to simple hill-climbing approach. (i) accuracy of the hill-climbing approach used over the complete network; (ii) accuracy of the hill-climbing approach after fixing the weakly connected nodes using our algorithm; (iii) accuracy of our combined pipeline using the genetic algorithm.

To provide an idea of the running times, Table 5 gives the empirical data from our experiments. The first two phases (detecting weaking connected vertices and recognizing dense regions) were run within seconds while the genetic algorithm was run for a fixed amount of time, and the top scoring candidates where chosen as shown in Table 3.

**Table 5:** Running times of the algorithm

Network	(i) 1-cut & degree 2 vertices (secs)	(ii) Quasi-clique predictions	(iii) Genetic algorithm
Yu	0:00:29	0:31:02	15:00:00
DIP	0:00:41	0:48:14	15:00:00
PAM	0:00:19	0:29:49	15:00:00
DM	0:00:24	1:18:03	15:00:00

Running times of our method on the model networks for each phase of the algorithm: (i) detecting 1-cut vertices and degree two vertices; (ii) predicting quasi-clique clusters; and (iii) running the genetic algorithm. We are reporting the average run time over three runs on each network. The implementation was tested on a Powermac G5 2 Ghz with 4 GB of RAM. Note that the genetic algorithm was run for a fixed amount of time, and the top scoring candidates achieved the quality as shown in Table 3. The times are shown in *hh.mm.ss *format.

### Inferring direct interactions from AP-MS experimental data

In order to apply our algorithm to biological data from AP-MS experiments, we used the raw data reported by Krogan *et al*. [18] for the 2186 putative interactions of *G_KroganFull_*. We only considered the subnetwork of tagged proteins, and further focussed our efforts on the analysis of 77 proteins that are well separated in the tag-induced subnetwork. Quantitative abundance estimates were derived from the peptide counts reported for each prey, and an experimentally derived connectivity matrix *M *was obtained after normalization (see Methods). Our full prediction algorithm was then run on the estimated connectivity matrix, resulting in a direct interaction graph prediction we call *G_Kim _*that consists of 164 interactions (See Additional File 2). The network *G_Kim _*was compared to *G_KroganHigh_*, the set of high-confidence interactions reported by Krogan *et al*., and to $G_{KroganHigh}^{Top}$, a subset of *G_KroganHigh _*consisting of the 164 (to compare against *G_Kim_*) most confident interactions they reported.

Both *G_KroganFull _*and *G_KroganHigh _*overlap *G_Kim _*quite substantially. These three sets of predictions were then compared against a set of high-quality binary interactions from *G_Yu_*. In Y2 H experiments, the interaction partners are separately screened using a genetic readout. Therefore, interactions from *G_Y u _*are believed to be direct, and thus used to test against the predictions from AP-MS data. On the other hand, these interactions may reflect only a subset of all direct interactions among the 77 proteins.

**Figure 3:**
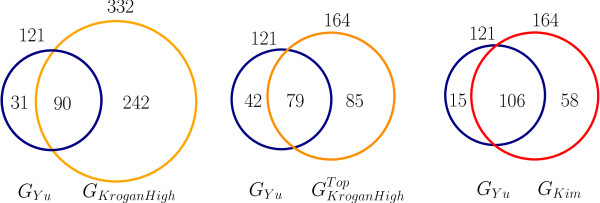
**Inferring direct interactions from actual AP-MS dataset**. Overlap between the Y2 H interaction network of Yu *et al*. and various AP-MS-based networks: (a) High-confidence set of interactions from Krogan *et al*. (b) Set of 164 highest scoring interactions from Krogan *et al*. (c) Set of 164 interactions predicted as direct interactions by our algorithms, based on the AP-MS data from Krogan *et al*.

As shown in Figure 3, our results show that the high-confidence AP-MS data *G_KroganHigh _*exhibited very little overlap with the direct binary interaction set *G_Yu_*. 72.6% of interactions in *G_KroganHigh _*is disjoint from *G_Y u_*, and 25% of *G_Yu _*remains undetected by *G_KroganHigh_*. Furthermore, even the top scoring set of interactions $G_{KroganHigh}^{Top}$ showed high discrepancy ratios against *G_Yu_*. In contrast, *G_Kim _*produced by our algorithm coincide with *G_Yu _*with better sensitivity and specificity. Given the crudeness of the method in translating the AP-MS data into a connectivity matrix, our algorithm has thus performed relatively well in predicting direct interactions from real AP-MS data.

## Discussion and conclusion

The approaches for determining bait-prey abundance remain in their infancy, and to date, no large-scale PPI networks have this type of quantitative data. As these approaches gain in accuracy, so will the results of our approach. Furthermore, as the sensitivity of AP-MS pipelines improves, the fraction of indirect interactions detected will also increase, thereby making the ability to distinguish them even more critical. In this paper, we lay the bases of modelling the indirect interactions in AP-MS experiments. We formulate the DIGCOM problem, which aims at distinguishing direct interactions from indirect ones, and provide a set of theoretical and heuristic approaches that are shown to be highly accurate on both biological PPI networks and simulated networks. Despite the unrealistic assumptions that should eventually be relaxed, our results show that the predicted set of interactions fits the experimental data reasonably well. In addition, applying our algorithms to a large-scale AP-MS data set from Krogan *et al*. results in predictions that overlap Y2H data approximately 35% more often than the equivalent number of top-scoring interactions reported by these authors.

The DIGCOM problems raise a number of challenging, yet fascinating computational and mathematical problems to investigate. Is the solution to the exact DIGCOM problem, if it exists, always unique? We suspect it is. What is the computational complexity of the exact and approximate DIGCOM problems?

We believe they are NP-hard, and possibly not even in NP. Are there types of graph substructures, other than those discussed here, that can be unambiguously inferred from *P_G_*? Are there special properties of PPI networks, other than the power-law degree distribution, of which an algorithm can take advantage to make more accurate predictions and/or provide approximation or probabilistic guarantees?

The modelling and algorithm proposed here is only a first step toward an accurate detection of direct interactions from AP-MS data. Several generalizations and improvements are worth investigating. First, the abundance of an interaction is not constant and needs to be modelled more accurately. Second, the strength of all physical interactions is non-uniform, and some interactions may be more prone to disruption by the affinity purification process than others. Given sufficient quantitative AP-MS data, one may study a generalization of the DIGCOM problem that aims at identifying not only the set of direct interactions, but also their individual strengths and abundances. While modelling these aspects is in theory possible, the amount and quality of experimental data required is currently unavailable, and the computational complexity of the resulting problems is likely to be daunting.

Perhaps a more significant limitation of our model is that all direct interactions are assumed to occur simultaneously, though it is clear that certain interactions are either mutually exclusive, or restricted to specific subcellular compartments or conditions. We are currently investigating approaches to decompose the observed network into a family of simultaneously occurring interactions in such a way that the observed interaction abundances are the sum of the direct and indirect interactions over all cell compartments and conditions. However, it is clear that complementary experimental data, such as comprehensive protein localization assays or cell cycle expression data, would be required to reduce the space of possible solutions in a biologically meaningful manner.

An additional assumption that may need to be relaxed is the independence of the edge failures, which may not hold in cases where the loss of an interaction between two proteins causes a significant destabilization of the larger complex they belong to. Unfortunately, in the presence of strong dependencies between edge failures, it becomes almost impossible to distinguish direct from indirect interactions. Nonetheless, it may be possible to at least identify complexes where such dependencies hold, by studying subsets of proteins for which the AP-MS data differs significantly from our model.

In conclusion, this paper opens the door to a number of fascinating modelling and algorithmic questions that will lead to important implications in systems biology. Any improvements in tackling these questions would take us one step further towards this goal.

## Methods

In this section, we describe the algorithmic details of our approach to the DIGCOM problem.

### Identification of weakly connected vertices

The algorithm to identify 1-cut vertices is trivial given Theorem 1: recursively find edges satisfying conditions in Theorem 1, and decompose the input matrix into two independent subproblems. On the other hand, the conditions in Theorem 2 yields Algorithm 1, which predicts the set of degree 2 vertices as well as the edges adjacent to them. It is easy to see that the algorithm to identify 1-cut vertices runs in time *O*(|*V*|^4^) and Algorithm 1 runs in time *O*(|*V*|^2^).

Note that the identification algorithm for 1-cut vertices allows us to *remove *these vertices, i.e., the corresponding rows and columns in the input matrix. This is possible due to the fact that removing a cut edge does not change the connectivity between any two nodes on the same side of the cut. On the other hand, we cannot simply remove degree-2 vertices without affecting the remaining entries in the matrix. Therefore, as shown in Algorithm 1, degree-2 vertices and their incident edges will be marked as such in the solution, but are not removed from the input matrix.

   **Predict degree-2 vertices**

   **Input**: Probability matrix *M *and vertex set *V*

   **Output**: Degree 2 vertices *V *^2 ^and their incident edges *E*^2^

   **foreach ***vertex s *∈ *V ***do**

      **if ***s satisfies conditions in Theorem 2 ***then**

         *V *^2 ^*← V *^2 ^∪ {*s*};

         *u, v ← *the two vertices with *M_s, u _*= *M_s, v _*= max{*M_s, t _*: ∀*t *∈ *V - *{*s*}};

         *E*^2 ^*← E*^2 ^∪ {(*s, u*), (*s, v*)};

      **end**

   **end**

   **Return ***V *^2 ^and *E*^2^

**Algorithm 1**: Prediction of degree 2 vertices

   **Detect dense clusters in the network**

   **Input**: Probability matrix *M*, and minimum cluster size *k*

   **Output**: Set of possible *k*-cliques in *G_direct_*.

   *t ← CliqueConn*(*k*) (the connectivity between two vertices in an *m*-clique; see Appendix)

   *G_t _← *(*V, E_t_*), where *E_t _*= {(*u, v*): *u, v *∈ *V *and *M*(*u, v*) ≥ *t*}

   **foreach ***connected component S *⊆ *V ***do**

      Find cliques of size *k *in *S*

   **end**

   **Return **cliques discovered.

**Algorithm 2**: Detecting dense clusters in the network

### Identification of densely connected regions

Densely connected regions are identified using a clique-cover algorithm (see Algorithm 2). We note that the algorithm guarantees to identify all cliques of size *k' *≥ *k *contained within *G_direct_*. However, sets of vertices that do not form a *k*-clique may also be reported, provided that they are sufficiently connected among themselves, possibly via vertices outside the set. However, for sufficiently large values of *k*, we found this to be a very rare occurrence. While finding cliques in a graph is a computationally intensive task in general, the construction of *G_t _*for large values of *k *creates few small connected components and leaves the remaining vertices isolated. Therefore, in practice, Algorithm 2 can be implemented to run in a reasonable amount of time.

### Cut-based genetic algorithm

The genetic algorithm aims at solving a generalization of the A-DIGCOM. First, we allow each edge (*u, v*) in the network to survive with a non-uniform probability $\hat{p}(u,\;v)$, instead of one probability $\hat{p}$ over all edges. Secondly, we assume that we are given two sets of edges *E_YES _*and *E_NO _*that indicate the set of edges that are guaranteed to be in the solution, and guaranteed not to be in the solution, respectively. This will later allow us to factor in the outcome of the previous sections. Therefore, the edges whose presence remains to be determined are $E_{MAYBE}=\bar{E_{YES}\cup E_{NO}}$.

#### Encoding of candidate solutions

To represent a candidate solution, we first create a hash table that maps each putative edge in *E_MAYBE _*to an integer. Each candidate is then encoded as a list of integers (edges). Edges in *E_YES_*, which are part of all solutions, are not explicitly listed, in order to save space. Since the networks we consider are sparse (|*E*| = *O*(|*V*|)), such an encoding technique significantly reduces the space requirements.

#### Initial population

The initial population of candidates is generated using a preferential attachment model [30] using the following observations: (i) The average connectivity of vertex *u*, $avgCon(u)=\frac{1}{\left|V\right|}\sum_{v\in V-\{u\}}M(u,v)$ is strongly positively correlated with the degree of *u *in *G_direct_*; (ii) the age of a vertex, measured by when the vertex was introduced to the graph, is positively correlated with the degree of the vertex. Therefore, during the generation of each candidate, we choose the next vertex to be added with probability proportional to its average connectivity. This results in a candidate solution where the degree of most vertices is likely to be close to the their true degree in *G_direct_*. Furthermore, in order to create candidates that are clustered similarly to the true direct interaction graph, we include the set of edges predicted by Algorithm 2 to each initial candidate.

#### Fitness function

The fitness of a candidate solution *G*, *fitness*(*G*) is obtained by first estimating the probability matrix *P_G _*using 500 Monte Carlo samples, and then counting the number of vertex pairs (*u, v*) whose estimated connectivity *P_G_*(*u, v*) is within the tolerance level *δ*, i.e., *M*(*u, v*) ± *δ *(See below for choosing the tolerance level *δ *).

#### Crossover

The crossover operation needs to hybridize two parent candidates to produce off springs preserving the good properties of the parents. This operation will be guided by a randomly chosen balanced cut *V *= *V*_1 _∪ *V*_2_. Let *G*_1 _and *G*_2 _denote the two parent networks and let *E*_1_(*G_i_*) and *E*_2_(*G_i_*) denote the edges of *G_i _*such that both endpoints lie in *V*_1 _and *V*_2_, respectively. Furthermore, let *E*_1,2_(*G_i_*) denote the edges of *G_i _*that crosses from *V*_1 _and *V*_2_. Mating *G*_1 _and *G*_2 _results in two children *G' *= (*V, E'*), and *G'*' = (*V, E'*') such that:

$$\begin{array}{l} E^{'}=E_{1}(G_{1})\cup E_{2}(G_{2})\cup (E_{1,2}(G_{1})\cap E_{1,2}(G_{2}))\\ E^{\prime \prime }=E_{2}(G_{1})\cup E_{1}(G_{2})\cup (E_{1,2}(G_{1})\cap E_{1,2}(G_{2})) \end{array}$$

While choosing a random cut as the crossover point is a reasonable strategy to construct a new pair of offsprings, our studies have shown that a planned strategy in choosing the crossover points results in better performance and less chance of premature convergence. In particular, if the crossover point is chosen at a dense cut in the parent networks, then the connectivity among vertices within each partition would be deteriorated significantly. This results in offsprings with much poorer fitness than their parents. On the other hand, if the parents are hybridized at a sparse cut, the connectivity among vertices within each partition are more localized. Therefore, crossover operations are best done by selecting sparse balanced cuts (|*V*_1_| ≈ |*V*_2_|). Finding sparse balanced cuts is a well-studied problem in combinatorial optimization, for which various approximation algorithms exist [38,39]. However, these algorithms assume that the graph itself, not the connectivity matrix *M*, is given as input. We therefore use a simple heuristic that avoids cutting through the dense regions of the network. To generate these sparse cuts, we contract each dense region identified in Algorithm 2 to a single vertex, and then generate weighted (by the number of vertices in each dense region) balanced partitioning of the vertices at random.

#### Mutation

In order to introduce variability to the population of candidates, a small number of edges (5 ~ 10%) are randomly inserted or deleted. Moreover, observe that the child network constructed as above may not remain connected. Aside from the random mutation, therefore, we employ a simple local search that greedily adds edges to keep the network connected.

#### Genetic algorithm parameter selection

The various parameters of the genetic algorithm were selected based on the resulting performance on the Yu *et al*. data set. Two main parameters that affect the performance significantly are the population size and the selection criteria. For selection criteria, we tested several different selection criteria by setting the probability of choosing a candidate as a parent. The best compromise between running time and accuracy was obtained using a population size of 500, and selection probability for a parent proportional to *fitness*(*c_i_*) - *minFit*, where *minFit *is the fitness of the worst candidate in the population (data not shown).

### Restricting the solution space

While our genetic algorithm offers a plausible method for the A-DIGCOM problem, one can reduce the size of the solution space, which typically results in faster convergence to better solutions, using the results in Theorem 1 and 2. First, recall that finding all cut edges decomposes the problem into independent subproblems on 2-edge-connected components. Second, the identification of degree-2 vertices defines two sets of edges *E_YES _*and *E_NO _*that constitute all putative edges incident to the identified degree-2 vertices. In other words, *E_MAY BE _*forms the subgraph of *G induced *by the set *V *^3+ ^of vertices with degree ≥ 3. Furthermore, observe that the edges in *E_Y ES _*form parallel paths between vertices in *V *^3+^. A classical result in network reliability (see Fact 2 in Appendix) suggests that these parallel paths can be merged into a single meta-edge whose reliability can be efficiently computed. To be more formal, let P (*u, v*) = {*P*_1_(*u, v*), *P*_2_(*u, v*), ..., *P_k_*(*u, v*)} be the set of paths between *u *and *v *in *E_Y ES_*. These paths can then be replaced by a single edge (*u, v*) with its survival probability $\hat{p}(u,v)=1-\Pi_{i=1...k}\left(1-{\hat{p}}^{\left|P_{i}(u,v)\right|}\right)$. By merging every set of parallel paths, we obtain a compact network over *V*^3+ ^that efficiently encodes the edges in *E_Y ES_*. Since our genetic algorithm handles the case where the edge survival probability is non-uniform, this compact encoding results in substantial gains in running time for estimating the fitness of the candidates, as well as in time and space requirements for handling large population sizes. In our applications, this allows us to remove approximately 70 ~ 75% of the original set of vertices.

### Randomized hill-climbing algorithm

For performance comparisons, we tested our algorithm against a simple randomized hill-climbing approach. In this approach, we start with a randomly chosen spanning tree *G*^1 ^of the vertex set *V*. At the *i^th ^*iteration, we first sample the connectivity probability $P_{G^{i}}$ of *G^i^*, using the Monte Carlo simulation. Then, we randomly pick a vertex pair *u, v *with probability proportional to

$$\frac{D(u,v)}{\sum_{\forall i,j\in V}D(i,j)},$$

where $D(u,v)=|M(u,v)-P_{G^{i}}(u,v)|$. If the selected pair *u, v *are connected by an edge in *G^i^*, but *M*(*u, v*) >$P_{G^{i}}$ (*u, v*), then we remove (*u, v*) from *G^i^*. On the other hand, if *u *and *v *are not connected by an edge, but *M*(*u, v*) <$P_{G^{i}}$ (*u, v*), then we add (*u, v*) to *G^i^*. We repeat this local optimization heuristic while making sure the candidate solution remains connected.

### **Choosing a tolerance level **δ **and handling numerical errors**

In order to deal with numerical errors from Monte Carlo sampling, we use a well-defined tolerance level *δ *as additive errors. Note that the sampling process for estimating the probability matrix *P_G _*is a binomial process, which, by the central limit theorem, is closely approximated by a normal distribution. The confidence interval is largest when the estimated probability is equal to 0.5, in which case we obtain a confidence interval of

$$\hat{p}\pm \delta =\hat{p}\pm \frac{z_{1-\alpha /2}}{2\sqrt{n}},$$

where $\hat{p}$ denotes the fraction of samples where the two vertices are connected after *n *samples, and *z*_1 - α*/*2 _is the z-value for desired level of confidence. Using this formula, we can conclude:

1. When *n *= 20000 (computation of our input matrix *M *from test networks), we obtain a 95% confidence interval of size at most 2·*δ *= 2·0.007 = 0.014.

2. When *n *= 500 (computation of the connectivity matrix for each candidate solution in our genetic algorithm), the 95% confidence interval is of size at most 2·*δ *= 2·0.04 = 0.08.

With the chosen tolerance level *δ*, we modify our algorithm as appropriate each time we compare two connectivity probabilities. For example, in Theorem 1, the first condition *P_G_*(*u, v*) = $\hat{p}$ is modified to$P_{G}(u,v)\in \left[\hat{p}-\delta ,\hat{p}+\delta \right]$; and in Theorem 2, we modify the first condition $P_{G}(s,t)<2\hat{p}-{\hat{p}}^{2}$ to $P_{G}(s,t)<2\hat{p}-{\hat{p}}^{2}+\delta$.

### Generation of scale-free networks

In order to generate artificial scale-free networks, we used two generation models: the preferential attachment model, and the duplication model. In the preferential attachment model, we evaluated the degree distribution of the two biological networks (*G_Y u _*and *G_DIP _*) and used the Barabási-Albert algorithm to construct a scale-free network with attachment factor 1.5 (each iteration adds a new vertex with 1 ~ 2 edges attached to existing vertices). In the duplication model, at each iteration, we randomly pick a vertex to duplicate with probability proportional to its degree and randomly drop the duplicated edges with probability at 0.5 in order to t the degree distributions and sparsity of biological networks.

### Calculation of connectivity matrix from peptide counts

The peptide count of a prey protein in an AP-MS experiment is the number of different peptides that have been observed by MS for that protein. We note that the peptide counts are biased towards preys with longer protein sequences, and to rectify this propensity, we normalized the abundance data by the protein sequence lengths to obtain the abundance ratios *R*(*i, j*). In order to turn the normalized abundance ratios into the connectivity matrix for our probabilistic graph model, we used a simple logistic function

$$\hat{M}(i,j)=\frac{1}{1+e^{-\frac{R\left(i,j\right)-\alpha }{\beta }}}\;,$$

where the parameters *α*, *β *are chosen so that the computed distribution of $\hat{p}$ fits the simulated connectivity distribution of *G_Y u_*, using a *χ*^2 ^test (*α *= 2.8921, *β *= -0.6318). In the cases where *R*(*i, j*) differ from *R*(*j, i*), we choose the average of the two entries to symmetrize the matrix.

## Competing interests

The authors declare that they have no competing interests.

## Authors' contributions

All authors jointly contributed to the design and analysis of the algorithms. EK and MB jointly contributed to the formulation of the problem, interpretation of the experiments, and preparation of the manuscript. Implementation and testing were done by EK. All authors have read and approved the final manuscript.

## Appendix

We start with two basic results that will prove useful when proving more complex theorems. Let *G*_1 _= (*V*_1_, *E*_1_) and *G*_2 _= (*V*_2_, *E*_2_) be two graphs. Then the following are true.

**Fact 1**. (Series composition) *Suppose V*_1 _∩ *V*_2 _= {*c*}, *and a new graph G is constructed by joining G*_1 _*and G*_2 _*at c. Then, for any s *∈ *V*_1 _*- *{*c*} *and for any *$t\in V_{2}-\{c\},P_{G}(s,t)=P_{G_{1}}(s,c)\cdot P_{G_{2}}(c,t)$.

**Fact 2**. (Parallel composition) *Suppose V*_1 _∩ *V*_2 _= {*s, t*}, *and a new graph G is constructed by joining G*_1 _*and G*_2 _*at s and t (possibly leading to parallel edges between s and t). Then*, $P_{G}(s,t)=P_{G_{1}}(s,t)+P_{G_{2}}(s,t)-P_{G_{1}}(s,t)\cdot P_{G_{2}}(s,t)$.

### Proof of Theorem 1

*Proof*. Necessity is trivial. For sufficiency, suppose the conditions (i) and (ii) hold, and (*u, v*) is not a cut edge. Then, to keep the graph connected, there must be an edge (*s, t*) ≠ (*u, v*) joining *V_u _*and *V_v_*. Since (*s, t*) is an edge, $p(s,\;t)\geq \hat{p}$. However, by assumption, we have $p(s,\;t)=p(s,\;u)\cdot \hat{p}\cdot p(v,t)<\hat{p}$, which is a contradiction.   □

### Proofs of Theorem 2

*Proof*. We prove each condition separately.

#### Condition (i) Low connectivity

Since *s *has degree 2, it becomes disconnected from the rest of the graph with probability ${\left(1-\hat{p}\right)}^{2}$. Thus, $P_{G}(s,\;t)\leq 1-{\left(1-\hat{p}\right)}^{2}=2\hat{p}-{\hat{p}}^{2}$. The equality can only hold if *P_G_*(*s, u*) = *P_G_*(*s, v*) = 1, which is impossible.

#### Condition (ii) Neighborhood

We first show *P_G_*(*s, u*) = *P_G_*(*s, v*). Let *P_E _*_**-**{(*s, u*)}_(*s, u*) denote the connectivity of *s *and *u *when edge (*s, u*) is removed from *E*. Then, we have:

$$\begin{array}{lll} P_{G}(s,u) & =\hat{p}+P_{E-\{(s,u)\}}(s,u)-\hat{p}\cdot P_{E-\{(s,u)\}}(s,u) & \left(\text{by Fact }2\right)\\ & =\hat{p}+\hat{p}\cdot P_{E-\{(s,u),(s,v)\}}(v,u)-{\hat{p}}^{2}\cdot P_{E-\{(s,u),(s,v)\}}(v,u) & \left(\text{by}\;\text{Fact}\;\;1\right)\\ & =\hat{p}+\hat{p}\cdot P_{E-\{(s,u),(s,v)\}}(u,v)-{\hat{p}}^{2}\cdot P_{E-\{(s,u),(s,v)\}}(u,v) & \\ & =\hat{p}+P_{E-\{(s,v)\}}(s,v)-\hat{p}\cdot P_{E-\{(s,v)\}}(s,v) & \\ & =P_{G}(s,v) & \end{array}$$

Next, we show that for any *t *∈ *V *- {*s, u, v*}, *P_G_*(*s, t*) <*P_G_*(*s, u*). For a subgraph *H *⊆ *G*, let *p*(*H*) denote the probability of observing *H *from a probabilistic graph *G*. We can write this probability as

$$p(H)={\hat{p}}^{\left|E(H)\right|}\cdot {\left(1-\hat{p}\right)}^{\left|E(G)|-|E(H)\right|}$$

Thus, for any two subgraphs *H_i_, H_j _*⊆ *G*, such that |*E*(*H_i_*)| = |*E*(*H_j_*)|, *p*(*H_i_*) = *p*(*H_j_*). Let ℋ(*s, t*) be the set of subgraphs of *G *where *s *and *t *are connected. We can then write the probabilities *P_G_*(*s, t*) and *P_G_*(*s, u*) as the sum of the probabilities of these subgraphs.

$$\begin{array}{l} P_{G}(s,t)=\sum_{H_{i}\in ℋ(s,t)}p(H_{i})\\ P_{G}(s,u)=\sum_{H_{j}\in ℋ(s,u)}p(H_{j}) \end{array}$$

Observe that these two sets of subgraphs may overlap:

$$\begin{array}{l} ℋ(s,t)=ℋ(s,t,u)\cup ℋ(s,t,\bar{u})\\ ℋ(s,u)=ℋ(s,t,u)\cup ℋ(s,\bar{t},u) \end{array}$$

where ℋ(*s, t, u*) is the set of subgraphs such that *s, t*, and *u *are all connected, while $ℋ(s,\;t\;,\bar{u})$ is the set of subgraphs where *s, t *belong to the same connected component that doesn't contain *u*. Thus, we now focus on $ℋ(s,\;t\;,\bar{u})$ and $ℋ(s,\;\bar{t},u)$. The subgraphs in $ℋ(s,\;\bar{t},u)$ can be partitioned as follows, depending on which component *v *belongs to:

(i) {*s, u, v*}, {*t*}: *s, u, v *belong to the same component, and *t *belongs to another component.

(ii) {*s, u*}, {*v*}, {*t*}: *s, u *belong to the same component, and each of *v *and *t *belongs to distinct components.

(iii) {*s, u*}, {*v, t*}: *s, u *belong to the same component, and *v, t *belong to another component.

It is easy to see that the cases (i) and (ii) are nonempty, since (*s, u*), (*s, v*) ∈ *E*(*G*).

Finally, consider the set of subgraphs in $ℋ(s,\;t\;,\bar{u})$. Let *H *be a subgraph in this set. Since *s *and *u *belong to distinct components, (*s, u*) ∉ *E*(*H*), and thus (*s, v*) ∈ *E*(*H*) in order to have *s *and *t *connected. We then make the following operation on *H *to construct *H'*: (1) remove (*s, v*) from *H*, and (2) insert (*s, u*). Then, *H' *now has *s *and *u *in the same component, and *v *and *t *in another component - and therefore, *H' *belongs the case (iii) of $ℋ(s,\;\bar{t},u)$. Furthermore, note that *H *and *H' *have the same number of edges. Therefore, there is a mapping from subgraphs in $ℋ(s,\;t\;,\bar{u})$ to subgraphs in case (iii) of $ℋ(s,\;\bar{t},u)$ with equal number of edges. Since the cases (i) and (ii) are nonempty, it follows that *P_G_*(*s, t*) <*P_G_*(*s, u*).

#### Condition (iii) "Triangle" inequality

Given the probabilistic graph *G*, we partition the subgraphs of *G *into four cases depending on the existence of the two edges (*s, u*) and (*s, v*).

• (*s, u*), (*s, v*) ∈ *E*: occurs with probability ${\hat{p}}^{2}$.

• (*s, u*) ∈ *E*, (*s, v*) ∉ *E*: occurs with probability $\hat{p}(1-\hat{p})$.

• (*s, u*) ∉ *E*, (*s, v*) ∈ *E*: occurs with probability$\hat{p}(1-\hat{p})$.

• (*s, u*), (*s, v*) ∉ *E*: occurs with probability${\left(1-\hat{p}\right)}^{2}$.

We can thus rewrite the probability *P_G_*(*s, t*) as follows.

(1) $$P_{G}(s,t)=\hat{p}\cdot (1-\hat{p})\cdot P_{G-\{s\}}(u,t)+\hat{p}\cdot (1-\hat{p})\cdot P_{G-\{s\}}(v,t)+{\hat{p}}^{2}\cdot P_{G-\{s\}}(u\text{ or }v,t)$$

where *P*_G - {*s*}_(*u *or *v, t*) denotes the probability that *u *or *v *is connected to *t *in the graph *G *- {*s*}. We write *P_G_*(*u, t*) and *P_G_*(*v, t*) similarly:

(2) $$P_{G}(u,t)=(1-{\hat{p}}^{2})\cdot P_{G-\{s\}}(u,t)+{\hat{p}}^{2}\cdot P_{G-\{s\}}(u\text{ or }v,t)$$

(3) $$P_{G}(v,t)\;=(1-{\hat{p}}^{2})\cdot P_{G-\{s\}}(v,t)+{\hat{p}}^{2}\cdot P_{G-\{s\}}(u\text{ or }v,t)$$

Subtracting (1) from (2), and (1) from (3) gives:

$$\begin{array}{l} P_{G}(u,t)-P_{G}(s,t)=(1-\hat{p})\cdot P_{G-\{s\}}(u,t)-\hat{p}(1-\hat{p})\cdot P_{G-\{s\}}(v,t)\\ P_{G}(v,t)-P_{G}(s,t)=(1-\hat{p})\cdot P_{G-\{s\}}(v,t)-\hat{p}(1-\hat{p})\cdot P_{G-\{s\}}(u,t) \end{array}$$

If $P_{G-\{s\}}(u,\;t)>\hat{p}\cdot P_{G-\{S\}}(v,\;t)$, it follows that *P_G_*(*u, t*) *> P_G_*(*s, t*). Otherwise, $P_{G-\{s\}}(u,\;t)\leq \hat{p}\cdot P_{G-\{s\}}(v,\;t)$ implies $\hat{p}\cdot P_{G-\{s\}}(v,\;t)<P_{G-\{s\}}(v,\;t)$, and it follows that *P_G_*(*v, t*) >*P_G_*(*s, t*).   □

### **Connectivity in an ***n***-clique**

Here we construct a formula to compute the connectivity of an *n*-clique *K_n_*, defined as CliqueConn(*n*) in Algorithm 2.

First, we recall a classical result in graph enumeration.

#### Lemma 1

[40,41]*Let γ_n _denote the number of connected simple graphs over n labeled vertices. We can count this number by the recurrence*.

$$\gamma_{n}=\{\begin{array}{ll} 1 & if\;n=1\\ 2^{\left(\begin{array}{c} n\\ 2 \end{array}\right)}-\frac{1}{n}\sum_{i=1}^{n-1}\left(i\left(\begin{array}{c} n\\ i \end{array}\right)\;\cdot \;2^{\left(\begin{array}{c} n-i\\ 2 \end{array}\right)}\cdot \gamma_{i}\right) & otherwise \end{array}$$

Now we show the formula for the case when $\hat{p}=\frac{1}{2}$, which is the value we used for our analyses. The formula for other values of $\hat{p}$ can be formulated using a similar proof.

#### Lemma 2

$$CliqueConn(n)=\sum_{i=2}^{n}\frac{\left(\begin{array}{c} n\\ i-2 \end{array}\right)\gamma_{i}}{2^{\left(\begin{array}{c} n\\ 2 \end{array}\right)-\left(\begin{array}{c} n-i\\ 2 \end{array}\right)}}$$

*Proof*. We write the probability as follows.

$$CliqueConn(n)=\frac{\sigma_{2}+\sigma_{3}+...+\sigma_{n}}{2^{\left(\begin{array}{c} n\\ 2 \end{array}\right)}}$$

where *σ_i _*denotes the number of subgraphs of *K_n _*of size *i *where two given vertices *u *and *v *are connected. To compute *σ_i_*, there are $\left(\begin{array}{l} n\\ i-2 \end{array}\right)$ ways to pick *i *- 2 vertices (in addition to *u *and *v*) for the connected components, and *γ_i _*ways to keep them all connected, and finally, $2\left(\begin{array}{l} n-i\\ 2 \end{array}\right)$ ways to assign edges among the remaining vertices. So we have

$$\sigma_{i}=\left(\begin{array}{c} n\\ i-2 \end{array}\right)\cdot \gamma_{i}\cdot 2^{\left(\begin{array}{c} n-i\\ 2 \end{array}\right)},$$

and finally, we have

$$CliqueConn(n)=\sum_{i=2}^{n}\frac{\left(\begin{array}{c} n\\ i-2 \end{array}\right)\gamma_{i}}{2^{\left(\begin{array}{c} n\\ 2 \end{array}\right)-\left(\begin{array}{c} n-i\\ 2 \end{array}\right)}}$$

   □

## Supplementary Material

Additional file 1 **ModelValidation**. Correlation plot of the connectivity from $P_{G_{Krogan\;High}}$ and normalized peptide counts from Krogan *et al*. Each data point corresponds to an interaction in *G_KroganFull _*- *G_KrogranHigh_*, many of which are expected to be indirect. x-axis: Connectivity entry in $P_{G_{Krogan\;High}}$, using $\hat{p}=0.5$. y-axis: Peptide count, normalized by protein lengths. The resulting linear regression showed a strong correlation (regression p-value 8.17 × 10^-11^, Student *t*-test), suggesting that many interactions in *G_KroganFull _*- *G_KrogranHigh _*are indirect and are the result of direct interactions in *G_KroganHigh_*. Click here for file

Additional file 2 **PredictedInteractions**. Set of direct interactions among 77 yeast proteins, predicted by our algorithm. The connectivity matrix is generated from normalized peptide counts, and then our algorithm is run to predict the direct interactions. Click here for file
