# Supplementary material for: Predicting direct protein interactions from affinity purification mass spectrometry data
Source: Algorithms Mol Biol. 2010 Oct 29;5:34. doi: 10.1186/1748-7188-5-34 (PMC2991326; doi:10.1186/1748-7188-5-34)

Additional file 1. – Model validation

Correlation plot of the connectivity from $P_{G_{KroganHigh}}$ and normalized peptide counts from Krogan *et al.* Each data point corresponds to an interaction in $G_{KroganFull} - G_{KrogranHigh}$, many of which are expected to be indirect. x-axis: connectivity entry in $P_{G_{KrogranHigh}}$, using $\hat{p} = 0.5$. y-axis: peptide count, normalized by protein lengths. The resulting linear regression showed a strong correlation (regression p-value 8.17×10^{-11}, Student t-test), suggesting that many interactions in $G_{KroganFull} - G_{KrogranHigh}$ are indirect and are the result of direct interactions in $G_{KroganHigh}$.

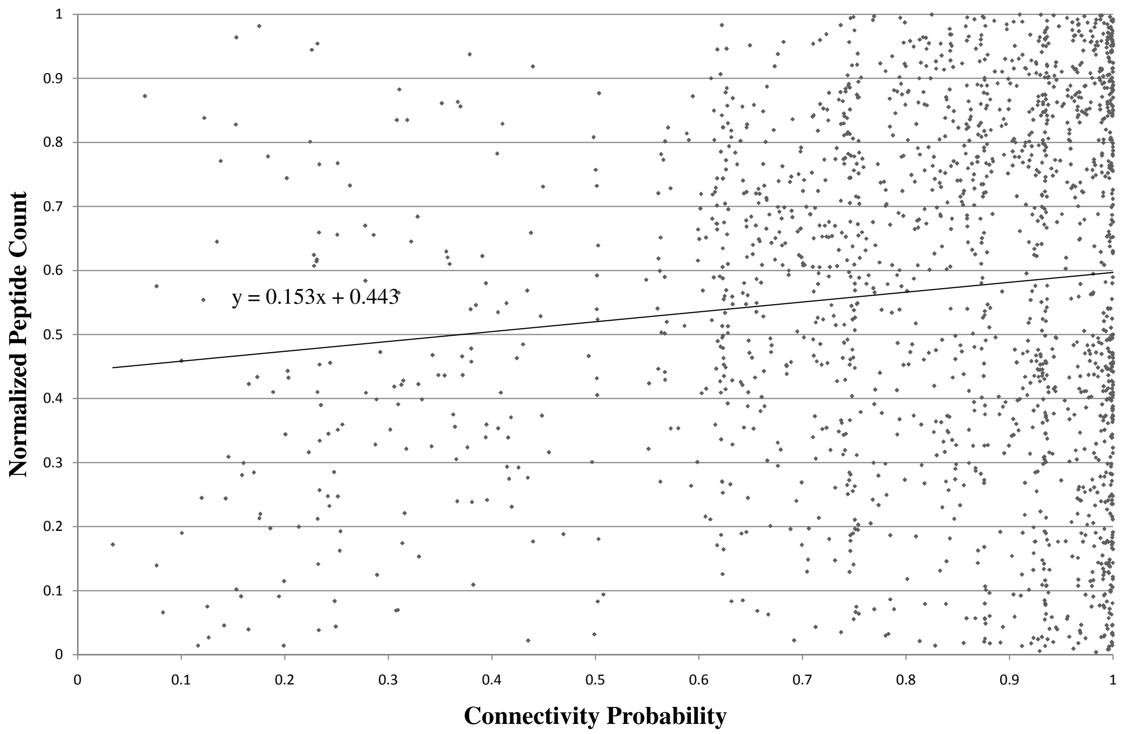

Supplement: Additional file 1 — ModelValidation. Correlation plot of the connectivity from PGKrogan High and normalized peptide counts from Krogan et al. Each data point corresponds to an interaction in GKroganFull - GKrogranHigh, many of which are expected to be indirect. x-axis: Connectivity entry in PGKrogan High, using p^=0.5. y-axis: Peptide count, normalized by protein lengths. The resulting linear regression showed a strong correlation (regression p-value 8.17 × 10^-11^, Student t-test), suggesting that many interactions in GKroganFull - GKrogranHigh are indirect and are the result of direct interactions in GKroganHigh. [file 1748-7188-5-34-S1.PDF]
